# Gender, rainfall endowment, and farmers’ heterogeneity in wheat trait preferences in Ethiopia

**DOI:** 10.1016/j.foodpol.2023.102584

**Published:** 2024-01

**Authors:** Hom N. Gartaula, Gebrelibanos Gebremariam, Moti Jaleta

**Affiliations:** a International Rice Research Institute (IRRI), New Delhi, India; b Mekelle University, Mekelle, Ethiopia; c International Maize and Wheat Improvement Center (CIMMYT), Addis Ababa, Ethiopia

**Keywords:** Wheat, Trait preferences, Gender, Rainfall, Multivariate Probit Model, Ethiopia

## Abstract

Wheat is a vital cereal crop for smallholders in Ethiopia. Despite over fifty years of research on wheat varietal development, consideration of gendered trait preferences in developing target product profiles for wheat breeding is limited. To address this gap, our study used sex-disaggregated survey data and historical rainfall trends from the major wheat-growing regions in Ethiopia. The findings indicated heterogeneity in trait preferences based on gender and rainfall endowment. Men respondents tended to prefer wheat traits with high straw yield and disease-resistance potential, while women showed a greater appreciation for wheat traits related to good taste and cooking quality. Farmers in high rainfall areas seemed to prioritize high straw yield and disease resistance traits, while those in low rainfall areas valued good adaptation traits more highly. Most of the correlation coefficients among the preferred traits were positive, indicating that farmers seek wheat varieties with traits that serve multiple purposes. Understanding men’s and women’s preferences and incorporating them in breeding and seed systems could contribute to the development of more targeted and effective wheat varieties that meet the diverse needs of men and women farmers in Ethiopia.

## Highlights

• We analyzed the wheat trait preferences of men and women farmers under varying levels of rainfall endowment.
• Both men and women valued high yield and good adaptation to climate variability most highly.
• Men exhibited a higher preference for high straw yield and disease resistance traits; women prioritized traits related to good taste and cooking quality.
• Households in rainfall-deficit areas tended to prioritize good adaptation, whereas those in rainfall-surplus areas prioritized disease resistance.

## 1 Introduction

Ethiopia is the largest wheat producer in sub-Saharan Africa (SSA), producing about 5.8 million tons of wheat annually on 2.1 million hectares (M ha) of land under rain-fed (1.7 M ha) and irrigated (0.4 M ha) systems (Csa, 2021, Hodson et al., 2020, Tadesse et al., 2022)**.** Over five million smallholders earn their living directly from wheat production (CSA, 2021); wheat is the second most important crop after maize in terms of production and contributes a substantial proportion to the national protein and calorie consumption (Badstue et al., 2022, Erenstein et al., 2022). Consumer demand for wheat is increasing due to growth in population, income, and urbanization. Although there is strong policy support from the government to expand wheat production and attain self-sufficiency in wheat consumption, there is limited information on the type of wheat varieties that both increase farm productivity and meet the demand for different traits among men and women consumers.

Wheat breeding programs face the challenge of developing varieties that satisfy the diverse interests of heterogeneous farmers and farming communities across different agroecologies and rainfall patterns. Farmers demand varieties that withstand biotic and abiotic stresses and perform better in terms of biomass and yield when climatic shocks happen. Depending on their different roles in the household, men and women farmers have different trait preferences for the wheat varieties that they grow or introduce to their farms. For example, women and marginalized farmers in India prefer wheat grain that makes better chapatis to wheat grain with yield-enhancing and risk-mitigating traits (Krishna and Veettil, 2022). In Nigeria, Teeken et al. (2021) found significant differences in men’s and women’s prioritization of cassava traits. To inform and guide wheat breeding, seed marketing, and extension programs on the dominant trait preferences of smallholders in diverse agroecological zones, it is important to understand how trait preferences vary by climatic factors (such as rainfall) and by the gender of the household members who decide on varietal choices.

Initiatives related to the development of seed systems of cereal crops primarily concentrate on the technical aspects of developing seed traits. However, they often overlook the social dynamics and demand-side factors within the seed system (Badstue et al., 2022, Barrett et al., 2022, Voss et al., 2021). For instance, the Green Revolution period, typically from 1967 to 1978, successfully increased calorie supplies to the global population and also helped reduce food prices, increase anthropometric outcomes, decrease agricultural intensification in forest areas, and reduce infant mortality (Barrett et al., 2022, Evenson and Gollin, 2003, Gollin et al., 2021). However, the focus on a few dominant cereals contributed to malnutrition, especially among rural farmers and urban consumers (Pingali, 2012, Welch and Graham, 2000). The Green Revolution also had unintended environmental effects and a highly skewed distribution of benefits towards some social groups, particularly men and better-off farmers (Barrett et al., 2022, Krishna and Veettil, 2022, Pingali, 2012). Owing to the absence of feedback mechanisms in the existing seed system, the diverse interests and preferences of men and women farmers have not been sufficiently addressed in wheat varietal development (Suri and Gartaula, 2023).

Gender differences in wheat trait preferences highlight the broader issue of social equity in agriculture. Studies have shown that women farmers often face structural constraints, such as limited access to resources and information, lack of decision-making power, and discriminatory social norms (Doss et al., 2015, Marenya et al., 2021a, Peterman et al., 2011, Quisumbing, 1996, Sekabira and Qaim, 2017). Doss (2002) found that men tended to have greater control over decisions related to cash crops and agricultural inputs, while women played a significant role in decisions related to household consumption and subsistence farming. Addressing these disparities in crop improvement and agricultural decision-making processes requires recognizing and valuing women’s knowledge, preferences, and contributions.

In recent years, development actors and policymakers have become more cognizant of the significance of gender equality and social inclusion in attaining development outcomes. Gender research has consistently highlighted the fact that attaining many objectives related to food security and nutrition appears to be influenced by women’s capacity to secure a favorable distribution of resources within households (Doss, 2013, Doss and Quisumbing, 2020, Fafchamps et al., 2009). However, the common model assumed, especially in the early economics literature, was the unitary household model (Becker, 1973). This model either assumed that all members of a household shared identical preferences, combined all their resources, and reached unanimous decisions, or presumed that one household member acted as decision-maker for the entire family (Alderman et al., 1995, Doss, 2013, Doss and Quisumbing, 2020, Quisumbing and Maluccio, 2003, Teklewold, 2023). Based on the unitary household model, numerous studies investigating trait preferences begin by considering the household as the primary unit of analysis. These studies commonly use the sex of the household head to represent gender differences (e.g., Kassie et al., 2017, Lunduka et al., 2012, Marenya et al., 2022, Marenya et al., 2021b).

However, the assumption of perfect cooperation and shared preferences may not accurately capture real-world scenarios (Alderman et al., 1995, Teklewold, 2023). A household typically consists of multiple individuals with distinct preferences, potentially leading to conflicts of interest in decision-making processes. As a result, alternative models, such as the collective household model, have been developed to account for the heterogeneity of preferences and degrees of bargaining power among household members (Agarwal, 1997, Fafchamps et al., 2009). Quisumbing and Maluccio (2003) examine the unitary and collective household models using data from Bangladesh, Ethiopia, Indonesia, and South Africa. In all these four countries, they find evidence that contradicts the validity of the unitary model as an accurate representation of household behavior. They demonstrate that the resources that individuals contribute to a marriage, such as human capital and personal assets, influence how resources are allocated within the household. This is primarily due to the influence of these resources on intrahousehold bargaining power (Agarwal, 1997).

Applying rigorous empirical methods in analyzing the wheat trait preferences of men and women farmers in Ethiopia, this paper contributes to the literature in two ways. First, we test the existence of gendered trait preferences between men and women respondents from the same household. This contributes to the limited literature showing the intrahousehold gender dynamics in wheat trait preferences, contributing to better product profile development. Although we were not able to test the unitary and collective household models,^1^ our gender-differentiated data within the same household enabled us to uncover gender disparities in trait preferences.

Second, there is an emerging recognition of the importance of weather-related variables in agricultural technology adoption and welfare implications. Amare et al. (2018) show that negative rainfall shocks have heterogeneous impacts on crop production across different agroecological zones in Nigeria. Similarly, Cui and Xie (2022) report that farmers in China adjust their crop planting dates based on weather conditions observed eight weeks before the actual planting period. In Ethiopia, Marenya et al. (2020) illustrate the heterogeneous adoption and impacts of sustainable agricultural intensification technologies in rainfall-surplus and rainfall-deficit plots. Studies have also examined the role of weather shocks on land-allocation decisions (Ahmed et al., 2022, He and Chen, 2022, Mu et al., 2018). Although these studies provide pertinent information to the literature about the role of weather-related shocks on many important aspects, to the best of our knowledge, no study has successfully tested the hypothesis that rainfall endowment differences (i.e., between households that receive a high amount and those that receive a low amount of rainfall) affect a rural household’s crop trait preferences. Ethiopia, where weather variability is high and rainfed agricultural activities constitute the single most important source of income for almost all rural households, provides a suitable case for this research. Investigating heterogeneous trait preferences among rural households that experience diverse rainfall patterns could help to identify policy options that are better tailored to the needs of socioeconomically diverse smallholders as well as to develop better-targeted breeding product profiles. In this context, we present how gender, rainfall endowment, and wheat trait preferences interact, and explore implications for agricultural research and development (R&D) in the developing country context.

The remainder of the paper is structured as follows. The next section outlines the data and methodology used in this study. The third section presents the estimation results and provides a comprehensive discussion of the findings. Finally, Section 4 concludes the paper by drawing key policy implications based on the results.

## 2 Data and methodology

### 2.1 Data source

Data for this research were obtained from a household survey conducted in Ethiopia between June and August 2021 as part of the Accelerating Genetic Gains in Maize and Wheat for Improved Livelihoods in Asia and Africa (AGG) project, which was led by the International Maize and Wheat Improvement Center (CIMMYT) in collaboration with national partners in Ethiopia. Using a structured questionnaire, data were collected from randomly sampled households in the three main wheat-producing regions (Amhara, Oromia and Southern Nations, Nationalities and Peoples, or SNNP) of Ethiopia. Sampling was done in three stages: We prioritized wheat-growing areas and identified districts growing wheat on land of more than 2,000 ha. We then identified the major wheat agroecologies within the selected districts. Following the proportionate random sampling technique, we selected 40 districts from the 6 wheat agroecologies identified. In each sample district, the major wheat-producing *kebeles* (the lowest administrative units) were identified, and 10–18 sample households were randomly selected from the household list available in each kebele. Following this procedure, we collected data from 1,088 sample households (Table 1). Trained enumerators proficient in the local languages were assigned to conduct the survey.

**Table 1:** Distribution of sample households by region and zone.

	Amhara		Oromia		SNNP		Total	
Agroecological zone	Kebeles	HHs	Kebeles	HHs	Kebeles	HHs	Kebeles	HHs
Tepid to cool humid mid-highlands	1	3	22	237	1	14	24	254
Cold to very cold humid sub-Afro-Alpine			1	7			1	7
Hot to warm moist lowlands	1	13					1	13
Tepid to cool moist mid-highlands	12	122	12	149	1	7	25	278
Hot to warm sub-humid lowlands			1	2			1	2
Tepid to cool semi-arid mid highlands			2	18			2	18
Tepid to cool sub-humid mid highlands	15	59	28	184	7	145	50	388
Tepid to cool sub-moist mid highlands	4	46	8	81	1	1	13	128
Total	33	243	74	678	10	167	117	1088

The survey collected detailed information on household socioeconomic characteristics, land and livestock holdings, income sources, non-agricultural household enterprises, household assets, shocks, seed sources, networks, and trait preferences, together with agricultural inputs, and crop production and disposition patterns. To select the traits for our study, we first compiled a list of 21 traits based on expert consultation and the existing literature on wheat preferences. We then proceeded to engage in group discussions and conducted a preliminary survey of the farmers to assess their preferences. Six traits (high grain yield, good adaptation, high straw yield, disease resistance, bold grain, good taste and cooking quality) emerged as resoundingly important among the participants. We then designed the final survey, incorporating these six wheat traits to obtain more focused and insightful responses from the households. A trait that was considered important by a respondent received a value of 1, and of 0 if not considered important.

We interviewed both a male member (typically the household head) and a female member (usually the wife) of the same household. These individuals, referred to as the principal male and principal female household members, were asked about their preferences for wheat traits, as well as about several other variables of interest. This approach enabled us not only to identify gender disparities in wheat trait preferences but also to control for other gender-related variables.

Additionally, we employed a global positioning system (GPS) to geo-reference both the households and their corresponding wheat plots. This allowed us to incorporate rainfall variables into our models and estimate wheat trait preferences based on the rainfall endowment. To generate these rainfall variables, we utilized data from the Climate Hazards Group InfraRed Precipitations with Stations (CHIRPS). CHIRPS is a comprehensive dataset that spans from 50°S to 50°N, encompassing all longitudes. It combines satellite imagery with in-situ station data at a resolution of 0.05° to create a gridded rainfall time series, providing valuable information for our analysis (Funk et al., 2015, Marenya et al., 2020, Michler et al., 2019). We generated monthly rainfall data from 2001 to 2021. We relied on boundaries on the 0.05° grid cells and took the average monthly rainfall for the month within the grid. We then estimated the season-level rainfall by aggregating the monthly data. From the rainfall data, we computed the annual total rainfall and rainfall shock indicators (discussed below). The amount of rainfall and the rainfall shock indicators for the year preceding the survey year were also calculated to capture the effect of rainfall abundance or stress experienced by the household in the previous year.

We estimated rainfall shocks as normalized deviations in a single season’s rainfall from the expected average seasonal rainfall over the 21 years between 2001 and 2021 (Marenya et al., 2020, Michler et al., 2019). The grid averages were mapped onto each household in the grid (based on farm GPS coordinates). The rainfall shock indicator was estimated as:

(1) $$R_{\mathrm{wt}}=\left|\frac{r_{\mathrm{wt}}-\bar{r_{w}}}{\sigma_{\mathrm{rw}}}\right|$$

The rainfall shock ${(R}_{\mathrm{wt}})$ was calculated for each household in a year t, where $r_{\mathrm{wt}}$ is the observed amount of rainfall for the whole agricultural season, ${\bar{r}}_{w}$ is the average seasonal rainfall for the household over the 21-year period, and $\sigma_{\mathrm{rw}}$ is the standard deviation of rainfall over the same period. A value of zero for the shock variable indicates that the household has not encountered any rainfall fluctuations. Conversely, a larger deviation from zero signifies a higher degree of rainfall variability or shock experienced by the household.

The measures of rainfall surplus ${(R}_{\mathrm{wt}}^{s})$ were also calculated as:

(2) $$R_{\mathrm{wt}}^{s}=\left\{\begin{array}{c} \left|\frac{r_{\mathrm{wt}}-\bar{r_{w}}}{\sigma_{\mathrm{rw}}}\right|\;if\;r_{\mathrm{wt}}>\bar{r_{w}}\\ 0\;\;\;\;otherwise \end{array}\right),$$

Similarly, the measures of rainfall deficit ${(R}_{\mathrm{wt}}^{d})$ were estimated as:

(3) $$R_{\mathrm{wt}}^{d}=\left\{\begin{array}{c} \left|\frac{r_{\mathrm{wt}}-\bar{r_{w}}}{\sigma_{\mathrm{rw}}}\right|\;if\;r_{\mathrm{wt}}<\bar{r_{w}}\\ 0\;\;\;\;otherwise \end{array}\right),$$

We used equations (2), (3) to categorize households into rainfall-surplus and rainfall-deficit groups and tested differences in wheat-trait preferences between the two groups, taking into account lagged rainfall shocks from the previous year.

### 2.2 Data analysis

Both men and women household members were asked if they thought that the six traits presented to them were important in their wheat varietal choices. To explore the driving factors behind selecting and combining various wheat traits, a multivariate probit model (MVP)^2^ estimator was used (Cappellari and Jenkins, 2003). Since respondents had the option to select multiple traits, our empirical approach had to account for interdependency among these traits and their correlations. So, the MVP estimator was deemed the most appropriate for this assessment. More importantly, the MVP estimator not only identifies the determinants of traits but also sheds light on the synergies and trade-offs among various traits. As a result, it has been extensively utilized in studies exploring interdependent technology adoptions (Gebremariam and Tesfaye, 2018, Kassie et al., 2015, Wainaina et al., 2016).

The observed outcome of a particular wheat trait can be modeled in a random utility framework (respondent’s utility). Consider the $i^{\mathrm{th}}$ respondent (man or woman) $\left(i=1,\cdots \cdots .N\right)$, who is facing a decision as to whether a given wheat trait is important in his/her future improved cultivar. Let $U_{0}$ represent the utility gained by the respondent when he/she decides not to take up a certain wheat trait, and let $U_{k}$ represent the utility gained by the respondent when he/she prefers the $k^{\mathrm{th}}$ wheat trait. A given respondent decides to seek the $k^{\mathrm{th}}$ wheat trait if the net benefit is greater than zero, that is $Y_{\mathrm{ik}}^{*}=U_{k}-U_{0}>0$. The net benefit ($Y_{\mathrm{ik}}^{*}$) that the respondent derives from the k^th^ wheat trait is, however, a latent variable that is determined by explanatory variables (X_i_) and unobserved characteristics (*µ_i_*). This can be modeled in the equation below.

(4) $$Y_{\mathrm{ik}}^{*}=X_{i}\beta_{i}+\mu_{i}$$

The MVP model is characterized by a set of binary wheat traits, that is equal to 1 if the i^th^ respondent prefers the wheat trait K,^3^ and zero otherwise, such that:

(5) $$Y_{k}=\left\{\begin{array}{c} 1\;if\;Y_{\mathrm{ik}}^{*}>0\\ 0\;otherwise \end{array}\right)\;K\left(T1,T2,T3,T4,T5,T6\right)$$

The MVP model allows for simultaneous preferences for different wheat traits. The error terms jointly follow a multivariate normal distribution (MVN) with zero conditional mean and variance normalized to unity where $\left(\mu_{A},\mu_{Y},\mu_{s}\right)\approx MVN\left(0,\Omega \right)$, and the symmetric covariance matrix Ω is given by:

(6) $$\Omega =\left[\begin{array}{cccccc} 1 & \rho_{T1T2} & \rho_{T1T3} & \rho_{T1T4} & \rho_{T1T5} & \rho_{T1T6}\\ \rho_{T2T1} & 1 & \rho_{T2T3} & \rho_{T2T4} & \rho_{T2T5} & \rho_{T2T6}\\ \rho_{T3T1} & \rho_{T3T2} & 1 & \rho_{T3T4} & \rho_{T3T5} & \rho_{T3T6}\\ \rho_{T4T1} & \rho_{T4T2} & \rho_{T4T3} & 1 & \rho_{T4T5} & \rho_{T4T6}\\ \rho_{T5T1} & \rho_{T5T2} & \rho_{T5T3} & \rho_{T5T4} & 1 & \rho_{T5T6}\\ \rho_{T6T1} & \rho_{T6T2} & \rho_{T6T3} & \rho_{T6T4} & \rho_{T6T5} & 1 \end{array}\right]$$

The Ω has a value of 1 on the leading diagonal and correlations $\rho_{\mathrm{jk}}=\rho_{\mathrm{kj}}$ as off-diagonal elements. The off-diagonal elements in the covariance matrix are of interest since they represent the unobserved correlations between the wheat traits. A positive and significant correlation between the error terms of wheat traits indicates complementarities or synergies. On the other hand, a negative correlation indicates substitutability or trade-offs between the traits (Börsch-Supan and Hajivassiliou, 1993, Cappellari and Jenkins, 2003, Gebremariam and Tesfaye, 2018).

## 3 Results and discussion

### 3.1 Descriptive statistics

Our sample comprises 1,088 households in total (984 men headed and 104 women headed households). To analyse gendered trait preferences, we tried to select both the principal man and the principal woman of the same household to ensure an equal representation of men and women within each sample household. However, we faced challenges when it came to finding typical male participants in the households headed by women. The male members in these households were either too young to interview or unavailable. Therefore, we were not able to capture data from men in the households headed by women. As a result, our analysis^4^ focused on the comparison between 984 men in male-headed households and 1,088 women in both men- and woman headed households.

Table 2 displays the summary statistics of our sample respondents, presented first at the household level and subsequently at the individual level (for men and then for women). The summary statistics indicate that high yield is predominantly the most preferred trait, as it was chosen by about 81 % of the respondents, both by men and women, at the household level. Good adaptation is the second most preferred trait, chosen by 74 % of households and by approximately 75 % of men and 73 % of women respondents. About 53 %, 54 % and 45 % of households, men respondents, and women respondents, respectively, selected the high straw yield trait, while disease resistance was chosen by approximately 68 % of households, 68 % of men respondents, and 64 % of women respondents. Bold grain, defined by large size and thick appearance, was selected by about 49 % of households, 49 % of men respondents, and 47 % of women respondents. Good taste and cooking quality were the least-preferred traits, selected by 27 % of households, 26 % of men respondents, and 32 % of women respondents, respectively.

**Table 2:** Descriptive statistics of variables used in the models.

Variables	Definition of variables	Household		Men		Women	
**Outcome variables**		Mean	Std	Mean	Std	Mean	Std
High yield	High yielding trait varieties (1 = if the respondent agrees this trait is important, 0 otherwise)	0.81	0.40	0.81	0.39	0.81	0.40
Good adaptation	Good adaptation trait varieties (1 = if the respondent agrees this trait is important, 0 otherwise)	0.74	0.44	0.75	0.43	0.73	0.45
High straw yield	High straw yield trait varieties (1 = if the respondent agrees this trait is important, 0 otherwise)	0.53	0.50	0.54	0.50	0.45	0.50
Disease resistance	Disease resistance trait varieties (1 = if the respondent agrees this trait is important, 0 otherwise)	0.68	0.47	0.68	0.47	0.64	0.50
Bold grain	Bold grain trait varieties (1 = if the respondent agrees this trait is important, 0 otherwise)	0.49	0.50	0.49	0.50	0.47	0.50
Good taste & cooking quality	Good taste & cooking quality trait varieties (1 = if the respondent agrees this trait is important, 0 otherwise)	0.27	0.44	0.26	0.44	0.32	0.47
**Explanatory Variables**							
Gender head	Gender of the head of the household (1 = male head, zero otherwise)	90.44	29.42	NA	NA	NA	NA
Age of respondent	Age of the respondent in the household (in years)	50.35	12.10	50.37	12.12	41.39	10.25
Education of respondent	Education level of the respondent (years of schooling)	5.47	5.67	6.05	5.66	1.23	2.61
Household size	Household size: total of all household members who live and share meals together in the household (persons)	6.06	2.14	NA	NA	NA	NA
Farm size	Total size of agricultural land cultivated by the household in hectares (ha)	1.88	1.27	NA	NA	NA	NA
Livestock owned	Tropical livestock unit (TLU) is used to normalize number of livestock to the equivalent of a cow whereby, cow = 1, sheep = 0.1, goat = 0.08, donkey = 0.5 respectively	6.99	4.70	NA	NA	NA	NA
Assets owned	Asset index calculated from twelve types of different assets owned by the household (index)	0.27	0.16	NA	NA	NA	NA
Mobile phone owned	Dummy variable. Self-reported = 1 if the household owned a mobile phone, 0 otherwise.	0.59	0.49	NA	NA	NA	NA
Improved wheat variety use	Dummy variable (1 = if the respondent indicated that he has cultivated improved wheat verities at least once in the past five years, 0 otherwise	0.49	0.50	0.49	0.50	0.38	0.49
Number of social networks in the village	The number of people (network) that the respondent has in the village that he can count on for help (in persons)	3.56	6.05	3.64	6.13	2.08	4.10
Contact with government extension worker	Dummy variable (1 = if the respondent had any contact with government extension agents, 0 otherwise)	0.81	0.39	0.82	0.39	0.63	0.48
Member of farmers’ group	Dummy variable (1 = if the respondent is a member of farmers’ group, 0 otherwise)	0.27	0.44	0.27	0.44	0.13	0.34
Member of savings and credit group	Dummy variable (1 = self-reported ability to access credit finance or savings for any purpose including farming whenever needed by the respondent, 0 otherwise.	0.11	0.31	0.11	0.31	0.06	0.24
Member of *Eddir*	Dummy variable (1 = if the respondent is a member of *Eddir*, 0 otherwise)	0.77	0.42	0.77	0.42	0.65	0.48
Member of *Equb*	Dummy variable (1 = if the respondent is a member of *Equb*, 0 otherwise)	0.03	0.18	0.03	0.18	0.03	0.16
Lagged rainfall amount received	Total amount of rainfall that the household received in the lagged year prior to the survey year (mm/year)	1235.34	214.39	NA	NA	NA	NA
Rainfall shock *(Index)*	Rainfall shock (index) that the household experiences. This is calculated by taking the absolute value of the difference between total rainfall of the growing season and the historical average of the 21 years rainfall divided by the standard deviation of rainfall in the 21 years period (Equation (3)	0.78	0.52	NA	NA	NA	NA
Rainfall surplus	Dummy variable (1 = if the household is in rainfall surplus area, 0 otherwise)	0.88	0.32	NA	NA	NA	NA
Oromia region	Dummy variable (1 = if the household is in Oromia region, 0 otherwise)	0.62	0.49	NA	NA	NA	NA
Amhara region	Dummy variable (1 = if the household is in Amhara region, 0 otherwise)	0.23	0.42	NA	NA	NA	NA
SNNP region	Dummy variable (1 = if the household is in SNNP region, 0 otherwise)	0.15	0.21	NA	NA	NA	NA
Observation		1088		994		1088	

NB: Our data comes from 984 men and 1088 women respondents from 1088 households (984 male- and 104 female-headed respectively. We use household and individual level variables in our analysis. NA indicates non-applicable.

About 90 % of the sample households were headed by men of about 50 years, whereas the average age of women respondents was 41 years. The average education level of the sampled household heads was approximately 5.5 school years. Among the individual respondents, men had an average education level of 6.09 school years, whereas women had an average of 1.23 school years. The average household size i.e., household members who lived and shared meals together in the same compound, was estimated at 6.10.

We included farm size (land owned), total livestock units (TLU) owned, asset index and mobile phone ownership as proxy measures of wealth. On average, the sample households owned 1.88 ha of land and 6.00 TLU of livestock. The asset index (estimated at 0.27, on average) was calculated from twelve different asset categories.

Institutional factors such as access to government extension, membership of a farmers’ group, and access to financial services were also included. For instance, 81 % of households had access to government extension services. Among the respondents, approximately 82 % of men, but only about 63 % of women, had access to government extension services. Access to savings and credit services was generally low in our sample, with only 11 % of households having access to credit. Among the respondents, 11 % of men and 6 % of women reported having access to savings and credit services. The average rainfall of the lagged year was estimated at 1,235 mm while the average rainfall shock index was estimated at 0.78.

### 3.2 Gendered wheat trait preferences

Analysis of gendered trait preferences reveals that there are both shared and distinct characteristics that appeal to men and women respondents. Table 3 shows that there was no significant difference in preferences between men and women for high yield, good adaptation, and bold grain. However, high yield and good adaptation were the most preferred traits by both men and women respondents (around 81 % and 74 %, respectively). This is consistent with the literature that finds that improved yield and reduced production risk are among the core expectations of smallholder farmers when selecting improved seed varieties (Macours, 2019) and of crop breeding and research programs in general (Krishna and Veettil, 2022).

**Table 3:** Trait preferences between men and women respondents.

Traits	Men (%)	Women (%)	Difference (%)
High yield	81.20	80.51	0.68
	(1.25)	(1.20)	(1.73)
Good adaptation	74.90	72.79	2.10
	(1.38)	(1.35)	(1.93)
High straw yield	54.17	45.31	8.85***
	(1.59)	(1.51)	(2.19)
Disease resistance	67.58	63.79	3.79*
	(1.49)	(1.54)	(2.09)
Bold grain	48.78	47.24	1.54
	(1.59)	(1.51)	(2.20)
Good cooking & taste quality	26.02	32.26	−6.24***
	(1.40)	(1.42)	(1.99)
Observations (N)	984	1088	

NB: Standard error in parenthesis. ***, **, and * shows significance level at 1%, 5%, and 10%, respectively.

We found, however, significant differences between men and women respondents in preferences for other traits. The underlying causes of gender differentiation in wheat trait preferences need to be further explored, and especially those relating to the cultivation of wheat for multiple purposes, such as for food and feed.^5^ High straw yield was an important attribute for varietal selection for 54 % of men but only for 45 % of women respondents, a statistically significant difference (p < 0.001). Crop residues are in high demand for livestock feed in Ethiopia (Jaleta et al., 2015), and livestock production is mainly the responsibility of men rather than women household members (Assan, 2014). In Ethiopia, crop residues are also used for construction (Jaleta et al., 2015, Tesfaye et al., 2004) and again, construction is mainly the responsibility of men. Therefore, it is not surprising that high straw yield is prioritized more by men respondents than by their women counterparts.

Differences in preference for disease resistance between men and women (68 % and 64 %, respectively) were weakly significant. More women than men respondents (32 % vs 26 %, respectively) preferred wheat varieties that had good taste and cooking qualities, a statistically significant difference. This finding is consistent with other studies that have found men to be more concerned with agronomic traits, while women prioritize traits relating to usage (Teeken et al., 2018, Tegbaru et al., 2020, Voss et al., 2021). Overall, we found that wheat trait preferences in the Ethiopian context were not gender neutral, at least for three of the six traits considered in this study.

### 3.3 Trait preference by rainfall endowment

Literature on the economics of agricultural technologies shows that underlying biophysical conditions such as rainfall stress explain much of the farm variation in the performance of agricultural technologies on land productivity (Abay et al., 2022, Amare et al., 2018, Hörner and Wollni, 2022, Marenya et al., 2020, Tanti et al., 2022). To investigate whether farmers’ trait preferences are affected by biophysical factors, we categorized households into rainfall-surplus and rainfall-deficit groups, based on historical rainfall data spanning over 21 years.

Men valued high yield more in rainfall-deficit than in rainfall-surplus areas (Table 4), possibly because low productivity in areas that experience rainfall shortage (Tesfaye et al., 2021) leads households to prioritize traits associated with improved yield under stress, such as high yield. Male respondents in rainfall-deficit areas showed a higher preference (85 %) for this trait than those in rainfall-surplus areas (73 %). Likewise, about 86 % of female respondents in rainfall-deficit areas and 72 % in rainfall-surplus areas valued good adaptation. Recent studies indicate that rural livelihoods in Ethiopia are highly susceptible to weather variability (Ahmed et al., 2022), and low rainfall is perceived as a significant challenge to crop production in the country. Agricultural outputs during lower-rainfall seasons are estimated to be nearly half those during higher-rainfall seasons (Kassie et al., 2013). Unsurprisingly, both men and women farmers in rainfall-deficit areas preferred traits with the potential for good climate adaptation compared with farmers in rainfall-surplus areas.

**Table 4:** Trait preferences difference by rainfall endowment and gender.

	Men respondents			Women respondents		
Traits	Rainfall deficit (%)	Rainfall surplus (%)	Difference(%)	Rainfall deficit (%)	Rainfall surplus (%)	Difference (%)
High yield	87.72	80.34	7.37*	85.83	79.86	5.98
	(3.08)	(1.34)	(3.88)	(3.90)	(1.29)	(3.83)
Good adaptation	85.09	73.36	11.52***	86.67	71.07	15.59***
	(3.35)	(1.49)	(4.30)	(3.11)	(1.46)	(4.28)
High straw yield	34.21	56.78	–22.57***	25.00	47.83	–22.83***
	(4.46)	(1.68)	(4.91)	(3.97)	(1.61)	(4.77)
Disease resistance	58.77	68.74	−9.96**	55.83	64.77	−8.94**
	(4.65)	(1.57)	(4.65)	(4.55)	(1.62)	(4.65)
Bold grain	41.23	49.77	−8.54*	44.17	47.62	−3.46
	(4.63)	(1.70)	(4.98)	(4.55)	(1.61)	(4.83)
Good taste & cooking quality	23.68	26.32	2.63	43.33	30.89	12.44***
	(3.99)	(1.49)	(4.37)	(4.54)	(1.49)	(4.51)

NB: Observations are from 984 men and 1088 women respondents. Standard error in parenthesis. ***, **, and * shows significance level at 1%, 5%, and 10%, respectively.

It was observed that wheat seed varieties that could produce a large quantity of straw (biomass) were valued more in rainfall-surplus areas than in rainfall-deficit areas by both men and women respondents. The difference was estimated at about 23 % and was significant at 1 % for both genders. The difference could be attributed to the higher livestock population in rainfall-surplus areas than in rainfall-deficit areas (Demeke et al., 2011). Moreover, given that crop residues serve as the primary source of animal feed in Ethiopia (Alkemade et al., 2013, Duncan et al., 2016, Jaleta et al., 2015), the preference for wheat varieties with a trait for high straw yield potential was more pronounced in rainfall-surplus areas than in rainfall-deficit areas for both men and women.

About 10 % more men respondents and 9 % more women respondents in rainfall-surplus areas prioritized disease resistance compared with men and women farmers in rainfall-deficit areas, a statistically significant difference. This is consistent with findings in the literature that crop diseases are more severe in areas where rainfall coverage is relatively higher than in low rainfall areas (Chiu et al., 2022, Ghini et al., 2011, Li et al., 2020). We found a significant difference of about 12 % in favor of good taste and cooking qualities only for women respondents in rainfall-deficit areas. Overall, as in the gender analysis, we found heterogeneous trait preferences by rainfall endowment.

### 3.4 Correlates of trait preferences

#### 3.4.1 Correlates of trait preferences (men respondents)

Table 5 displays the MVP estimates of the correlates of men respondents for the six wheat traits. The likelihood ratio test provides evidence supporting the rejection of the null hypothesis of zero correlation between the error terms (p < 0.01), consistent with the significant pairwise correlation coefficient between the error terms of the six wheat trait preference equations presented in Table 7.

**Table 5:** Multivariate probit estimation of the determinants of wheat trait preferences for men respondents.

	(1)	(2)	(3)	(4)	(5)	(6)
Explanatory variables	High yield	Good adaptation	High straw yield	Disease resistant	Bold grain	Good taste and cooking quality
Age of man respondent *(years)*	−0.002	−0.011*	−0.009	−0.005	−0.005	−0.001
	(0.007)	(0.006)	(0.006)	(0.006)	(0.005)	(0.006)
Age of woman respondent *(years)*	−0.006	0.002	0.006	0.010	0.008	−0.001
	(0.008)	(0.007)	(0.006)	(0.006)	(0.006)	(0.007)
Education of man respondent *(years of schooling)*	−0.023**	−0.022**	0.002	−0.013	−0.004	0.005
	(0.010)	(0.009)	(0.009)	(0.009)	(0.008)	(0.009)
Education of woman respondent *(years of schooling)*	−0.007	−0.016	−0.012	0.013	−0.031*	0.012
	(0.021)	(0.019)	(0.018)	(0.019)	(0.018)	(0.018)
Household size *(persons)*	−0.007	−0.036	−0.032	−0.010	−0.059***	0.016
	(0.025)	(0.024)	(0.023)	(0.023)	(0.022)	(0.023)
Farm size *(ha)*	0.039	0.084*	0.006	−0.061	0.082**	−0.011
	(0.049)	(0.045)	(0.042)	(0.042)	(0.040)	(0.042)
Livestock owned (*TLU*)	0.012	0.012	0.027**	−0.016	−0.001	0.005
	(0.013)	(0.012)	(0.011)	(0.011)	(0.010)	(0.011)
Assets owned *(Index)*	1.450***	0.347	−0.052	0.037	0.415	0.479
	(0.451)	(0.401)	(0.373)	(0.387)	(0.353)	(0.385)
Household owns mobile phone *(1 = yes)*	0.123	0.330***	−0.559***	0.371***	0.099	0.358***
	(0.133)	(0.121)	(0.116)	(0.120)	(0.109)	(0.118)
Household used improved wheat variety *(1 = if man respondent said ‘yes’)*	−0.065	0.112	0.045	0.117	0.101	−0.166
	(0.158)	(0.144)	(0.132)	(0.135)	(0.125)	(0.140)
Household used improved wheat variety *(1 = if woman respondent said ‘yes’)*	−0.381**	−0.543***	−0.078	0.291**	−0.114	0.266*
	(0.166)	(0.152)	(0.138)	(0.146)	(0.134)	(0.149)
Number of social networks man respondent has in the village	−0.004	0.015	−0.012	0.018	0.003	−0.009
	(0.011)	(0.011)	(0.010)	(0.012)	(0.010)	(0.011)
Number of social networks woman respondent has in the village	−0.005	0.000	0.091***	0.017	0.003	0.016
	(0.018)	(0.016)	(0.019)	(0.020)	(0.014)	(0.015)
Man respondent has had contact with a government extension worker *(1 = yes)*	0.003	−0.094	−0.755***	0.005	−0.090	−0.476***
	(0.185)	(0.169)	(0.154)	(0.154)	(0.148)	(0.166)
Woman respondent has had contact with a government extension worker *(1 = yes)*	−0.469***	−0.376***	0.237**	0.354***	0.570***	0.599***
	(0.154)	(0.133)	(0.119)	(0.119)	(0.113)	(0.134)
Man respondent is a member of a farmers’ group (1) *= yes)*	0.083	−0.070	−0.029	0.372***	0.102	0.362***
	(0.137)	(0.121)	(0.113)	(0.123)	(0.107)	(0.113)
Woman respondent is a member of a farmers’ group (1) *= yes)*	0.066	0.155	0.292*	0.033	−0.194	−0.154
	(0.176)	(0.160)	(0.150)	(0.161)	(0.142)	(0.149)
Man respondent is member of savings and credit group (1) *= yes)*	1.155***	0.454**	0.421**	−0.189	0.453***	0.026
	(0.289)	(0.196)	(0.178)	(0.177)	(0.172)	(0.177)
Woman respondent is member of savings and credit group (1) *= yes)*	−0.086	−0.263	−0.124	−0.001	0.504**	0.354*
	(0.284)	(0.216)	(0.213)	(0.210)	(0.206)	(0.205)
Man respondent is member of *Eddir (1 = yes)*	0.733***	0.427***	0.274*	−0.480***	−0.198	0.155
	(0.168)	(0.154)	(0.155)	(0.162)	(0.143)	(0.153)
Woman respondent is member of *Eddir (1 = yes)*	−0.149	0.351***	−0.095	0.095	−0.411***	−0.154
	(0.152)	(0.130)	(0.124)	(0.128)	(0.119)	(0.124)
Man respondent is member of *Equb (1 = yes)*	0.903**	0.240	0.213	0.767**	0.251	−0.184
	(0.452)	(0.314)	(0.288)	(0.311)	(0.259)	(0.287)
Woman respondent is member of *Equb (1 = yes)*	−0.673*	−0.400	0.041	−0.012	0.000	−0.045
	(0.380)	(0.322)	(0.322)	(0.342)	(0.289)	(0.319)
Lagged rainfall amount received *(mm/year)*	0.001**	0.000	−0.002***	0.000	−0.000*	−0.001**
	(0.000)	(0.000)	(0.000)	(0.000)	(0.000)	(0.000)
Rainfall shock *(Index)*	−0.249**	0.014	0.372***	0.238**	0.149	0.356***
	(0.118)	(0.112)	(0.108)	(0.108)	(0.098)	(0.106)
Rainfall surplus *(dummy: 1 = yes)*	−0.464**	−0.380**	0.496***	0.072	−0.009	−0.268
	(0.202)	(0.183)	(0.170)	(0.153)	(0.147)	(0.163)
Oromia region *(dummy: 1 = yes)*	0.725***	0.497***	−0.964***	1.043***	0.815***	−0.171
	(0.196)	(0.181)	(0.187)	(0.185)	(0.171)	(0.186)
Amhara region *(dummy: 1 = yes)*	0.023	0.298	−1.274***	0.153	0.077	0.614***
	(0.198)	(0.186)	(0.195)	(0.191)	(0.178)	(0.189)
Constant	0.240	0.336	2.874***	−1.052**	−0.121	−0.528
	(0.581)	(0.531)	(0.507)	(0.511)	(0.480)	(0.527)
Observations	984	984	984	984	984	984

Note: Standard errors in parentheses. Significance levels correspond with ***p <.01 for 1 %, **p <.05 for 5 % and, *p <.1 for 10 %.

**Table 6:** Multivariate probit estimation of the determinants of wheat trait preferences for women respondents.

	(1)	(2)	(3)	(4)	(5)	(6)
Variables	High yield	Good adaptation	High straw yield	Disease resistant	Bold grain	Good taste and cooking quality
Sex of household head *(dummy, 1 = male)*	0.297	0.606***	0.149	−0.009	0.098	0.062
	(0.187)	(0.176)	(0.164)	(0.173)	(0.160)	(0.169)
Age of man respondent *(years)*	−0.009	−0.011*	−0.006	0.006	−0.012**	−0.008
	(0.00624)	(0.0058)	(0.005)	(0.006)	(0.00532)	(0.006)
Age of woman respondent *(years)*	0.004	0.0103	−0.001	−0.00287	0.009	0.009
	(0.007)	(0.007)	(0.006)	(0.006)	(0.006)	(0.006)
Education of man respondent *(years of schooling)*	−0.008	−0.011	0.008	−0.014*	0.009	−0.002
	(0.01)	(0.009)	(0.008)	(0.009)	(0.008)	(0.009)
Education of woman respondent *(years of schooling)*	0.0023	−0.021	−0.047***	0.003	−0.041**	0.007
	(0.020)	(0.018)	(0.018)	(0.019)	(0.018)	(0.018)
Household size *(persons)*	−0.028	−0.039*	−0.015	0.011	−0.05**	−0.039*
	(0.024)	(0.022)	(0.021)	(0.022)	(0.021)	(0.022)
Farm size *(ha)*	−0.001	0.056	0.060	0.022	0.061*	−0.031
	(0.044)	(0.042)	(0.037)	(0.039)	(0.036)	(0.040)
Livestock owned (*TLU*)	0.01	0.012	0.012	−0.019*	−0.009	−0.014
	(0.011)	(0.011)	(0.010)	(0.010)	(0.009)	(0.010)
Assets owned *(Index)*	1.864***	0.548	−0.609*	−0.196	0.422	0.020
	(0.423)	(0.373)	(0.349)	(0.360)	(0.337)	(0.361)
Household owns mobile phone *(1 = yes)*	−0.019	0.184	−0.353***	0.497***	0.030	0.529***
	(0.121)	(0.112)	(0.106)	(0.113)	(0.104)	(0.110)
Household used improved wheat variety *(1 = if men respondent said ‘yes’)*	−0.056	−0.191	0.135	0.008	0.121	−0.210
	(0.151)	(0.137)	(0.129)	(0.131)	(0.125)	(0.137)
Household used improved wheat variety *(1 = if women respondent said ‘yes’)*	−0.374**	−0.322**	−0.315**	0.471***	0.042	0.192
	(0.160)	(0.143)	(0.136)	(0.140)	(0.132)	(0.144)
Number of social networks man respondent has in the village	−0.001	0.009	−0.016	0.002	0.008	0.008
	(0.010)	(0.011)	(0.010)	(0.009)	(0.009)	(0.009)
Number of social networks woman respondent has in the village	0.005	0.034*	0.038**	0.006	−0.011	−0.013
	(0.016)	(0.018)	(0.016)	(0.014)	(0.014)	(0.014)
Man respondent has had contact with government extension worker *(1 = yes)*	−0.210	−0.281*	−0.515***	0.068	−0.242*	−0.702***
	(0.170)	(0.156)	(0.141)	(0.146)	(0.141)	(0.153)
Woman respondent has had contact with government extension worker *(1 = yes)*	−0.258*	−0.131	0.384***	0.356***	0.463***	0.789***
	(0.140)	(0.125)	(0.115)	(0.116)	(0.112)	(0.128)
Man respondent is member of farmers’ group (1) *= yes)*	0.176	−0.059	−0.141	0.331***	0.001	0.251**
	(0.129)	(0.115)	(0.107)	(0.117)	(0.106)	(0.111)
Woman respondent is member of farmers’ group (1) *= yes)*	−0.0918	0.366**	−0.263*	−0.058	−0.031	−0.004
	(0.158)	(0.153)	(0.139)	(0.149)	(0.136)	(0.139)
Man respondent is member of savings and credit group (1) *= yes)*	0.574**	0.133	0.217	−0.262	0.129	0.286*
	(0.226)	(0.182)	(0.168)	(0.170)	(0.169)	(0.171)
Woman respondent is member of savings and credit group (1) *= yes)*	−0.125	0.014	−0.007	0.236	0.465**	0.067
	(0.240)	(0.210)	(0.193)	(0.193)	(0.194)	(0.202)
Man respondent is member of *Eddir (1 = yes)*	0.512***	0.619***	0.115	−0.665***	−0.170	0.035
	(0.156)	(0.146)	(0.143)	(0.153)	(0.138)	(0.145)
Woman respondent is member of *Eddir (1 = yes)*	0.034	0.134	−0.112	0.145	−0.276**	0.079
	(0.139)	(0.125)	(0.118)	(0.124)	(0.115)	(0.122)
Man respondent is member of *Equb (1 = yes)*	0.692*	−0.07	0.242	0.145	0.306	−0.295
	(0.379)	(0.300)	(0.265)	(0.276)	(0.262)	(0.275)
Woman respondent is member of *Equb (1 = yes)*	−0.409	−0.0871	0.271	0.319	−0.188	−0.0945
	(0.339)	(0.319)	(0.288)	(0.315)	(0.293)	(0.303)
Lagged rainfall amount received *(mm/year)*	0.001**	0.001	−0.001***	0.000	−0.000	0.000
	(0.000)	(0.001)	(0.000)	(0.000)	(0.000)	(0.000)
Rainfall shock *(Index)*	−0.030	−0.020	0.402***	0.164	0.188**	0.300***
	(0.110)	(0.104)	(0.097)	(0.102)	(0.093)	(0.098)
Rainfall surplus *(dummy: 1 = yes)*	−0.269	−0.508***	0.557***	−0.0237	−0.177	−0.555***
	(0.184)	(0.173)	(0.155)	(0.145)	(0.141)	(0.149)
Oromia region *(dummy: 1 = yes)*	0.566***	0.345**	−0.237	0.922***	0.833***	0.233
	(0.177)	(0.169)	(0.164)	(0.171)	(0.163)	(0.173)
Amhara region *(dummy: 1 = yes)*	0.290	0.125	−0.388**	0.094	0.302*	0.989***
	(0.182)	(0.174)	(0.172)	(0.177)	(0.172)	(0.178)
Constant	−0.201	−0.258	1.114**	−0.972**	−0.150	−0.731
	(0.534)	(0.500)	(0.465)	(0.485)	(0.455)	(0.488)
Observations	1,088	1,088	1,088	1,088	1,088	1,088

Note: Standard errors in parentheses. Significance levels correspond with ***p <.01 for 1 %, **p <.05 for 5 % and, *p <.1 for 10 %.

**Table 7:** Correlation coefficient of error terms obtained from the MVP model estimation of the traits.

	Men			Women	
Traits	Correlation coefficient	Std error		Correlation coefficient	Std error
High yield and Good adaptation	0.448***	(0.066)		0.422***	(0.062)
High yield and High straw yield	0.009	(0.060)		0.003	(0.057)
High yield and Disease resistance	−0.025	(0.063)		−0.166**	(0.061)
High yield and Bold grain	0.076	(0.060)		0.007	(0.057)
High yield and Good taste & cooking quality	0.083	(0.066)		0.118**	(0.065)
Good adaptation and High straw yield	0.002	(0.056)		0.098**	(0.055)
Good adaptation and Disease resistance	−0.025	(0.059)		−0.037	(0.058)
Good adaptation and Bold grain	0.065	(0.056)		0.111**	(0.055)
Good adaptation and Good taste & cooking quality	0.122**	(0.059)		0.057	(0.058)
High straw yield and Disease resistance	0.019	(0.057)		−0.019	(0.056)
High straw yield and Bold grain	0.351***	(0.055)		0.146***	(0.053)
High straw yield and Good taste & cooking quality	0.287***	(0.061)		−0.113**	(0.056)
Disease resistance and Bold grain	0.233***	(0.056)		0.205***	(0.054)
Disease resistance and Good taste & cooking quality	0.020	(0.063)		0.197***	(0.058)
Bold grain and Good taste & cooking quality	0.325***	(0.059)		0.346***	(0.056)

Standard errors in parentheses. Significance levels correspond with ***p <.01 for 1 %, **p <.05 for 5 % and, *p <.1 for 10 %.

We incorporated the characteristics of both men and women respondents in our models to account for the probability that members of a household may influence each other in their trait preference decisions. We also provided results for the correlates of trait preferences for both men and women, along with their respective characteristics, in the Supplemental Tables. Household-level variables that measure endowments (e.g., family size, land size), accessibility (e.g., social networks, contact with extension workers), and experience (e.g., characteristics of the respondent such as age and education level) were included as control variables in our models. Older men tended to place less value on traits related to good adaptation to climate change, consistent with studies that found a negative relationship between age and agricultural technology adoption (Gebremariam and Tesfaye, 2018), including trait preferences (Marenya et al., 2022).

As expected, for men respondents, livestock ownership (measured in total livestock units (TLU) was positively correlated with wheat traits indicative of high straw yield. We included an asset index based on ownership of durable goods and agricultural machinery and on housing conditions to control for the expected effects of wealth on trait preferences. The asset index was positively associated with men respondents’ demand for high yield traits, similar to results on the positive effect of asset ownership in promoting the adoption of improved agricultural technologies (Arslan et al., 2014). Access to mobile phones significantly increased the likelihood of prioritizing good adaptation, disease resistance, and good taste and cooking quality traits. These results indicate the importance of access to information in helping farmers overcome barriers to gaining knowledge about new varieties.

To account for rainfall, we included the previous year’s rainfall, the lagged year’s rainfall shocks as given in Eq. (1), and whether the household experienced a rainfall surplus or deficit in the lagged year. The results show that rainfall extremes (variability) matter. Farmers who experienced a high amount of rainfall during the previous cropping season were more likely to opt for high-yielding wheat varieties. The rainfall shock indicator (a proxy for erratic weather conditions) was found to be negatively associated with the high yield trait but positively associated with the other wheat traits. This suggests that smallholder farmers who operate under erratic weather conditions demand wheat varieties that can help them reduce production risks. Households who experienced a shortage of rainfall during the previous cropping season are more likely to demand varieties that produce high yield and have good adaptation features compared with households who experienced surplus rainfall. On the other hand, the high straw yield is preferred more in rainfall-surplus areas than in rainfall-deficit areas.

#### 3.4.2 Correlates of traits preferences (women respondents)

The coefficients obtained from the MVP model for wheat trait preferences among women respondents are presented in Table 6. As with the men respondents, the likelihood ratio test provided evidence that enabled us to reject the null hypothesis of zero correlation between the error terms (p < 0.01).

The asset index demonstrates a positive correlation with the high yield trait, but a negative correlation with the high straw yield trait for women respondents. Additionally, women respondents in households owning a mobile phone are more likely to opt for traits such as disease resistance and good taste and cooking quality.

The data reveal a positive association between social networks, specifically the number of people in a village that women respondents interact with, and wheat traits such as good adaptation and high straw yield. Having access to savings and credit is positively associated with the bold grain trait for women respondents. These findings are consistent with existing agricultural technology adoption literature that emphasizes the importance of social networks and credit access (Gebremariam and Tesfaye, 2018, Ndiritu et al., 2014, Teklewold et al., 2020, Tesfaye et al., 2021).

Our analysis also revealed that rainfall shocks had a significant association with most of the trait preferences among women. The rainfall shock indicator, serving as a proxy for unpredictable weather conditions, shows a positive correlation with traits such as high straw yield, bold grain, and good taste and cooking quality for women respondents. Smallholder women farmers operating under unpredictable weather conditions seek wheat seed varieties that can help mitigate production risks. Additionally, we observed that the previous year’s rainfall was positively associated with the high yield trait but negatively associated with the high straw yield trait. Overall, these rainfall shock results emphasize the significance of weather-related variables in the design of gendered improved seed varieties. The existing literature also highlights how such weather conditions impact the varietal adoption and welfare of households (Ahmed et al., 2022, Amare et al., 2018, Marenya et al., 2020).

### 3.5 Complementarities among the wheat traits

The MVP estimator generates correlation matrices that enable the observation of synergies and trade-offs in preferences for the various wheat traits. The results of these correlations are presented in Table 7. In mixed crop-livestock farming systems, crop production has multiple purposes (crop production for human consumption and straw production for animal feed, for example). Thus, improved wheat varieties with a single or particular trait might be insufficient from a farmer’s perspective. As expected, significant correlations were observed between the preferences for several of the improved wheat traits, suggesting that farmers opted for a wheat seed that would serve them for multiple purposes.

Most of the estimated correlation coefficients among the wheat traits are positive and significant, indicating complementarities among the traits. A negative relationship between the wheat traits suggests that respondents perceive trade-offs among them. However, a negative correlation may sometimes simply indicate a difference in suitability based on the biophysical farming conditions and the perception of the respondents (Gebremariam and Tesfaye, 2018, Wainaina et al., 2016). We found a positive correlation between the high yield and good adaptation traits for both men and women. This outcome aligns with our finding that the majority of men and women respondents (about 81 % and 74 %, respectively) favored these two traits the most. Studies also show that the yield enhancement and risk reduction aspects of improved seed are the most important features in rural households’ seed demand (Badstue et al., 2022, Macours, 2019, Marenya et al., 2021b; 2022).

Contrary to our expectations, for women respondents, high yield had a significant trade-off with disease-resistance. Likewise, preference for high yield was also significantly complementary with good taste and cooking quality for women respondents. Good climate adaptation was also significantly complementary with both high straw yield and bold grain for women respondents. For men respondents, good adaptation was a trait that could be complementary with good taste and cooking quality.

The estimated positive correlation between high straw yield and bold grain for both men and women respondents suggests that households view the two traits as complementary, consistent with the experimental literature on wheat production, which also found that wheat varieties that produced bold gain may also produce a relatively high amount of straw (Hussain et al., 2016). The finding that bold grain also has a positive correlation with disease resistance and good taste and cooking quality for both men and women has important policy implications since the implementation of a policy promoting one of the traits may result in spillover effects on others.

## 4 Conclusions and policy implications

To develop wheat varieties that are suitable for end users, including men and women farmers, it is important to consider differences in preferences for traits that feed into the target product profiles. This “demand-driven” breeding would help increase the adoption of improved wheat varieties, which is crucial for increasing agricultural productivity and reducing poverty in a more equitable and sustainable manner. In this study, we have analyzed the factors that influence the gendered trait preferences of wheat farming households, using survey data collected from 1,088 households in Ethiopia. We used a MVP model and simple test statistics to elicit the gendered determinants of trait preferences as well as rainfall endowment-based trait preferences. Our results show that men and women farmers in Ethiopia are very keen to change their existing wheat varieties in favor of more promising wheat traits. This supports growing R&D prioritization and policy interest in up-scaling agricultural intensification and crop improvements among smallholder farmers. Specifically, farmers prefer wheat varieties that increase yield and have good adaptation to climate variability.

The study identified the presence of significant heterogeneity in preferences for wheat varietal traits. Our gender-disaggregated results show that wheat trait preferences are not gender neutral; rather, there is a significant difference in trait preferences between men and women household members. Men are more interested in wheat traits such as high straw yield and resistance to disease, whereas women value good taste and cooking quality more highly than men. In rainfall-deficit areas, where less rainfall coverage is usually a major production challenge, traits such as good adaptation to climate variability are prioritized more than in rainfall-surplus areas. In areas with surplus rainfall, where disease manifestations and rainfall lodging might be the main production challenges, disease resistance is valued more than in rainfall-deficit areas. Relationships among the trait preferences show some strong complementarities (e.g., high yield and good climate adaptation). Hence, breeding programs and seed systems should strategically deliver wheat varieties that address the multiple interests of both men and women farmers in their varietal choices.

Our findings have important implications for the development of target product profiles. These results suggest that wheat improvement programs should continue to focus on breeding for high yield and good adaptation to climate change as these two traits are the most valued by both men and women farmers. However, other traits are also important. We suggest that the development of new wheat varieties, while paying attention to the role of gender and rainfall endowments, should also use multi-criteria evaluations to ensure that complementary traits are optimally weighted in variety-release criteria.

In situations where farmers showed a strong preference for wheat traits with the potential for adapting to climate variability, it is worth implementing insurance programs that protect farmers against the negative effects of rainfall shocks. Especially in situations where wheat traits for high yield and adaptation to climate variability are not available together, the provision of crop insurance bundles with high-yielding varieties could potentially stabilize farmers’ income and reduce the risk associated with adverse weather conditions. By mitigating the financial losses caused by such shocks, crop insurance could provide farmers with more confidence and stability and enable them to adopt high-yielding varieties and complementary inputs that improve production and enhance household food security.

Based on the findings of this study, some policy recommendations could be made to enhance wheat-based cropping systems in Ethiopia. Given the high preference for wheat varieties with high yield potential and good adaptation to climate variability shown by both men and women farmers, policy makers and development practitioners should prioritize the dissemination of improved wheat varieties that possess these characteristics. This could be achieved through targeted extension services, farmers’ field schools, and the provision of affordable access to quality seed. In addition, the divergent trait preferences between men and women farmers call for developing gender-specific strategies in varietal development and seed delivery systems that cater to the specific needs and preferences of each group. Moreover, in addressing the observed influence of rainfall patterns on trait preferences, policies should focus on strengthening farmers’ understanding of the relationship between crop traits and local environmental conditions. This could be achieved through capacity building and tailored advisory services for men and women farmers on climate-smart agricultural extension programs, where farmers are equipped with the knowledge and skills to adapt their farming practices to the changing climatic conditions. Furthermore, providing insurance against rainfall shocks could be a promising policy avenue to reducing farmers’ risk in choosing high-yielding varieties and improving their adaptive capacities.

Finally, we would like to address some limitations of our study. Firstly, we focused on only six wheat traits that are commonly explored in trait preference research and were identified through pre-survey results and expert discussions to analyze preferences. However, it is important to note that there are numerous other traits that could be considered in future studies. Including a wider range of traits in the analysis would provide a more comprehensive understanding of preferences. Secondly, although we examined gendered differences in trait preferences and incorporated significant variables that were disaggregated by sex, there are still several variables that were not disaggregated by gender, such as asset ownership and the distinct activities undertaken by men and women in the households. Having this level of disaggregation would have allowed for more precise results and interpretations, considering the gender dynamics at play. Additionally, while it can be challenging and has limitations, conducting trait preference assessments through framed experiments would have provided more informative insights. Despite not utilizing such approaches in this study, our findings remain relevant and can offer valuable policy recommendations.

## CRediT authorship contribution statement

**Hom N. Gartaula:** Conceptualization, Methodology, Validation, Investigation, Writing – original draft, Writing – review & editing, Supervision, Project administration. **Gebrelibanos Gebremariam:** Conceptualization, Data curation, Formal analysis, Investigation, Methodology, Writing – original draft, Writing – review & editing. **Moti Jaleta:** Conceptualization, Data curation, Formal analysis, Investigation, Methodology, Validation, Writing – original draft, Writing – review & editing.

## Declaration of competing interest

The authors declare that they have no known competing financial interests or personal relationships that could have appeared to influence the work reported in this paper.

## Footnotes

1 We could not test the unitary and collective models because we lack specific disaggregated data for key variables such as asset ownership and bargaining power.

2 We employed the Stata software package to estimate our MVP models. Additionally, we conducted separate MVP models for men and women respondents, using 100 simulations and robust standard error techniques.

3 T1=high yield, T2=good adaptation, T3=high straw yield, T4=disease resistance T5=bold grain and T6=good taste and cooking quality.

4 As a robustness check, we have included the results for the balanced sample of 984 men and women in male-headed households in the appendix. The results are similar to the results presented in Table 3 (see Table S1 for the balanced gender trait preference results and Table S2 for the rainfall endowment results in the supplementary tables). The MVP results for women in male-headed households are also presented in Table S3.

5 See also Voss et al., (2021), on the issue of multi-purpose improved maize varieties.

## References

[b0005] Abay K.A., Abay M.H., Amare M., Berhane G., Aynekulu E. (2022). Mismatch between Soil Nutrient Deficiencies and Fertilizer Applications: Implications for Yield Responses in Ethiopia. Agricultural Economics.

[b0010] Agarwal B. (1997). *“*Bargaining” and Gender Relations: Within and beyond the Household. Feminist Economics.

[b0015] Ahmed M.H., Tesfaye W.M., Gassmann F. (2022). Early Growing Season Weather Variation, Expectation Formation and Agricultural Land Allocation Decisions in Ethiopia. Journal of Agricultural Economics.

[b0020] Alderman H., Chiappori P.A., Haddad L., Hoddinott J., Kanbur R. (1995). Unitary versus Collective Models of the Household: Is It Time to Shift the Burden of Proof?. World Bank Research Observer.

[b0025] Alkemade R., Reid R.S., Van Den Berg M., De Leeuw J., Jeuken M. (2013). Assessing the Impacts of Livestock Production on Biodiversity in Rangeland Ecosystems. Proceedings of the National Academy of Sciences of the United States of America.

[b0030] Amare M., Jensen N.D., Shiferaw B., Cissé J.D. (2018). Rainfall Shocks and Agricultural Productivity: Implication for Rural Household Consumption. Agricultural Systems.

[b0035] Arslan A., McCarthy N., Lipper L., Asfaw S., Cattaneo A. (2014). Adoption and Intensity of Adoption of Conservation Farming Practices in Zambia. Agriculture, Ecosystems and Environment.

[b0040] Assan N. (2014). Gender Disparities in Livestock Production and Their Implication for Livestock Productivity in Africa. Scientific Journal of Animal Science.

[b0045] Badstue L., Krishna V.V., Jaleta M., Gartaula H., Erenstein O. (2022). Gender, Wheat Trait Preferences, and Innovation Uptake: Lessons from Ethiopia and India. Ooutlook on Agriculture.

[b0050] Barrett C.B., Benton T., Fanzo J., Herrero M., Nelson R.J. (2022). Springer Nature Sustainable Development Goals Series.

[b0055] Becker G. (1981).

[b0060] Börsch-Supan A., Hajivassiliou V.A. (1993). Smooth Unbiased Multivariate Probability Simulators for Maximum Likelihood Estimation of Limited Dependent Variable Models. Journal of Econometrics.

[b0065] Cappellari L., Jenkins S.P. (2003). Multivariate Probit Regression Using Simulated Maximum Likelihood. The Stata Journal.

[b0070] Chiu M.C., Chen C.L., Chen C.W., Lin H.J. (2022). Weather Fluctuation Can Override the Effects of Integrated Nutrient Management on Fungal Disease Incidence in the Rice Fields in Taiwan. Scientific Reports.

[b0075] Csa (2021). Central Statistical Agency (CSA): Report on Area and Production of Majr. Crops..

[b0080] Cui X., Xie W. (2022). Adapting Agriculture to Climate Change through Growing Season Adjustments: Evidence from Corn in China. American Journal of Agricultural Economics.

[b0085] Demeke A.B., Keil A., Zeller M. (2011). Using Panel Data to Estimate the Effect of Rainfall Shocks on Smallholders Food Security and Vulnerability in Rural Ethiopia. Climatic Change.

[b0095] Doss C.R. (2002). Men’s Crops? Women’s Crops? The Gender Patterns of Cropping in Ghana. World Development.

[b0100] Doss C. (2013). Intrahousehold Bargaining and Resource Allocation in Developing Countries. World Bank Research Observer.

[b0105] Doss C., Kovarik C., Peterman A., Quisumbing A., van den Bold M. (2015). Gender Inequalities in Ownership and Control of Land in Africa: Myth and Reality..

[b0110] Doss C.R., Quisumbing A.R. (2020). Understanding Rural Household Behavior: Beyond Boserup and Becker. Agricultural Economics.

[b0115] Duncan A.J., Bachewe F., Mekonnen K., Valbuena D., Rachier G., Lule D., Bahta M., Erenstein O. (2016). Crop Residue Allocation to Livestock Feed, Soil Improvement and Other Uses along a Productivity Gradient in Eastern Africa. Agriculture, Ecosystems and Environment.

[b0120] Erenstein, Olaf, Moti Jaleta, Khondoker Abdul Mottaleb, Kai Sonder, Jason Donovan, and Hans-Joachim Braun. 2022. Global Trends in Wheat Production, Consumption and Trade. Pp. 47–66 in Matthew P. Reynolds and Hans-Joachim Braun *“Wheat Improvement: Food Security in a Changing Climate.”* Springer Cham.

[b0125] Evenson R., Gollin D. (2003). Assessing the Impact of the Green Revolution. Science.

[b0130] Fafchamps M., Kebede B., Quisumbing A.R. (2009). Intrahousehold Welfare in Rural Ethiopia. Oxford Bulletin of Economics and Statistics.

[b0135] Funk C., Peterson P., Landsfeld M., Pedreros D., Verdin J., Shukla S., Husak G., Rowland J., Harrison L., Hoell A., Michaelsen J. (2015). The Climate Hazards Infrared Precipitation with Stations - A New Environmental Record for Monitoring Extremes. Scientific Data.

[b0140] Gebremariam G., Tesfaye W. (2018). The Heterogeneous Effect of Shocks on Agricultural Innovations Adoption: Microeconometric Evidence from Rural Ethiopia. Food Policy.

[b0145] Ghini R., Bettiol W., Hamada E. (2011). Diseases in Tropical and Plantation Crops as Affected by Climate Changes: Current Knowledge and Perspectives. Plant Pathology.

[b0150] Gollin D., Hansen C.W., Wingender A.M. (2021). Two Blades of Grass: The Impact of the Green Revolution. Journal of Political Economy.

[b0155] He X.i., Chen Z. (2022). Weather, Cropland Expansion, and Deforestation in Ethiopia. Journal of Environmental Economics and Management.

[b0160] Hodson D.P., Jaleta M., Tesfaye K., Yirga C., Beyene H., Kilian A., Carling J., Disasa T., Alemu S.K., Daba T., Alemayehu Y., Badebo A., Abeyo B., Erenstein O. (2020). Ethiopia’s Transforming Wheat Landscape: Tracking Variety Use through DNA Fingerprinting. Scientific Reports.

[b0165] Hörner D., Wollni M. (2022). Does Integrated Soil Fertility Management Increase Returns to Land and Labor?: Plot-Level Evidence from Ethiopia. Agricultural Economics.

[b0170] Hussain M., Waqas-Ul-Haq M., Farooq S., Jabran K., Farroq M. (2016). The Impact of Seed Priming and Row Spacing on the Productivity of Different Cultivars of Irrigated Wheat under Early Season Drought. Experimental Agriculture.

[b0175] Jaleta M., Kassie M., Erenstein O. (2015). Determinants of Maize Stover Utilization as Feed, Fuel and Soil Amendment in Mixed Crop-Livestock Systems, Ethiopia. Agricultural Systems.

[b0180] Kassie G.T., Abdulai A., Greene W.H., Shiferaw B., Abate T., Tarekegne A., Sutcliffe C. (2017). Modeling Preference and Willingness to Pay for Drought Tolerance (DT) in Maize in Rural Zimbabwe. World Development.

[b0185] Kassie B.T., Hengsdijk H., Rötter R., Kahiluoto H., Asseng S., Van Ittersum M. (2013). Adapting to Climate Variability and Change: Experiences from Cereal-Based Farming in the Central Rift and Kobo Valleys. Ethiopia. *Environmental Management*.

[b0190] Kassie M., Teklewold H., Jaleta M., Marenya P., Erenstein O. (2015). Understanding the Adoption of a Portfolio of Sustainable Intensification Practices in Eastern and Southern Africa. Land Use Policy.

[b0195] Krishna V.V., Veettil P.C. (2022). Gender, Caste, and Heterogeneous Farmer Preferences for Wheat Varietal Traits in Rural India. PLoS ONE.

[b0205] Li H.R., Xiang H.M., Zhong J.W., Ren X.Q., Wei H., Zhang J.E., Qiu Yuan X.u., Zhao B.L. (2020). Acid Rain Increases Impact of Rice Blast on Crop Health via Inhibition of Resistance Enzymes. Plants.

[b0210] Lunduka R., Fisher M., Snapp S. (2012). Could Farmer Interest in a Diversity of Seed Attributes Explain Adoption Plateaus for Modern Maize Varieties in Malawi?. Food Policy.

[b0215] Macours K. (2019). Farmers Demand and the Traits and Diffusion of Agricultural Innovations in Developing Countries. Annual Review of Resource Economics.

[b0220] Marenya P., Gebremariam G., Jaleta M., Rahut D.B. (2020). Sustainable Intensification among Smallholder Maize Farmers in Ethiopia: Adoption and Impacts under Rainfall and Unobserved Heterogeneity. Food Policy.

[b0225] Marenya P., Gebremariam G., Rahut D.B. (2021). Performance of Women-Managed Plots Compared to Men-Managed Plots among Smallholder Maize Farmers in Western and Central Ethiopia. Journal of Applied Economics.

[b0230] Marenya P.P., Wanyama R., Alemu S., Woyengo V. (2021). Trait Preference Trade-Offs among Maize Farmers in Western Kenya. Heliyon.

[b0235] Marenya P., Wanyama R., Alemu S., Westengen O., Jaleta M. (2022). Maize Variety Preferences among Smallholder Farmers in Ethiopia: Implications for Demand-Led Breeding and Seed Sector Development. PLoS ONE.

[b0240] Michler J.D., Baylis K., Arends-Kuenning M., Mazvimavi K. (2019). Conservation Agriculture and Climate Resilience. Journal of Environmental Economics and Management.

[b0245] Mu J.E., McCarl B.A., Sleeter B., Abatzoglou J.T., Zhang H. (2018). Adaptation with Climate Uncertainty: An Examination of Agricultural Land Use in the United States. Land Use Policy.

[b0250] Ndiritu W., Kassie M., Shiferaw B. (2014). Are There Systematic Gender Differences in the Adoption of Sustainable Agricultural Intensification Practices?. Evidence from Kenya. *Food Policy*.

[b0255] Peterman A., Quisumbing A., Behrman J., Nkonya E. (2011). Understanding the Complexities Surrounding Gender Differences in Agricultural Productivity in Nigeria and Uganda. Journal of Development Studies.

[b0260] Pingali, Prabhu. 2012. Green Revolution: Impacts, Limits, Andthe Path Ahead. *Proceedings of the National Academy of Sciences of the United States of America,* 109(31): 12302–8. 10.1073/pnas.0912953109 PMC3411969 22826253

[b0265] Quisumbing A.R. (1996). Male-Female Differences in Agricultural Productivity: Methodological Issues and Empirical Evidence. World Development.

[b0270] Quisumbing A.R., Maluccio J.A. (2003). Resources at Marriage and Intrahousehold Allocation: Evidence from Bangladesh, Ethiopia, Indonesia, and South Africa. Oxford Bulletin of Economics and Statistics.

[b0275] Sekabira H., Qaim M. (2017). Can Mobile Phones Improve Gender Equality and Nutrition? Panel Data Evidence from Farm Households in Uganda. Food Policy.

[b0285] Suri B., Gartaula H. (2023). Examining the Wheat Seed Delivery System in Bihar, India, Using a Gender Lens. Gender, Technology and Development.

[b0290] Tadesse W., Zegeye H., Debele T., Kassa D. (2022). Wheat Production and Breeding in Ethiopia : Retrospect and Prospects. Crop Breeding Genetics and Genomics.

[b0295] Tanti P.C., Jena P.R., Aryal J.P., Rahut D.B. (2022). Role of Institutional Factors in Climate-smart Technology Adoption in Agriculture: Evidence from an Eastern Indian State. Environmental Challenges.

[b0300] Teeken B., Olaosebikan O., Haleegoah J., Oladejo E., Madu T., Bello A., Parkes C.E., Kulakow P., Kirscht H., Tufan H. (2018). Cassava Trait Preferences of Men and Women Farmers in Nigeria: Implications for Breeding. Economic Botany.

[b0305] Teeken B., Garner E., Agbona A., Balogun I. (2021). Beyond ‘Women’s Traits’: Exploring How Gender, Social Difference, and Household Characteristics Influence Trait Preferences. Frontiers in Sustainable Food Systems.

[b0310] Tegbaru A., Menkir A., Baco M., Idrisou L., Sissoko D., Eyitayo A.O., Abate T., Tahirou A. (2020). Addressing Gendered Varietal and Trait Preferences in West African Maize. World Development Perspectives.

[b0315] Teklewold H. (2023). Understanding Gender Differences on the Choices of a Portfolio of Climate-Smart Agricultural Practices in Sub-Saharan Africa. World Development Perspectives.

[b0320] Teklewold H., Adam R., Marenya P. (2020). What Explains the Gender Differences in the Adoption of Multiple Maize Varieties? Empirical Evidence from Uganda and Tanzania. World Development Perspectives.

[b0325] Tesfaye W., Blalock G., Tirivayi N. (2021). Climate-Smart Innovations and Rural Poverty in Ethiopia: Exploring Impacts and Pathways. American Journal of Agricultural Economics.

[b0330] Tesfaye A.M., Kitaw D.G., Kahliew A., Ayalew L., Mebratu T. (2004).

[b0335] Voss R.C., Donovan J., Rutsaert P., Cairns J.E. (2021). Gender Inclusivity through Maize Breeding in Africa: A Review of the Issues and Options for Future Engagement. Outlook on Agriculture.

[b0340] Wainaina P., Tongruksawattana S., Qaim M. (2016). Tradeoffs and Complementarities in the Adoption of Improved Seeds, Fertilizer, and Natural Resource Management Technologies in Kenya. Agricultural Economics.

[b0345] Welch R.M., Graham R.D. (2000). A New Paradigm for World Agriculture: Productive, Sustainable, Nutritious, Healthful Food Systems. Food and Nutrition Bulletin.

