# Supplementary material for: Gender, rainfall endowment, and farmers’ heterogeneity in wheat trait preferences in Ethiopia
Source: Food Policy. 2024 Jan;122:102584. doi: 10.1016/j.foodpol.2023.102584 (PMC10830438; doi:10.1016/j.foodpol.2023.102584)
Supplement: Supplementary data 1 [file mmc1.docx]

**Gender, rainfall endowment, and heterogeneity in farmers’ wheat trait preferences in Ethiopia**

**Supplementary tables**

The supplementary material presents additional analyses that offer the interested reader further reference and context for the results presented in the main paper.

**List of Tables**

[Table S1: Trait preferences between men and women respondents (Balanced 984 men and women respondents) 2](#_Toc152683341)

[Table S2: Trait preferences difference by rainfall endowment and gender (Balanced sample) 3](#_Toc152683342)

[Table S3: Multivariate probit estimation of the determinants of wheat traits for women respondents (Balanced sample) 4](#_Toc152683343)

[Table S4: Multivariate probit estimation of the determinants of wheat traits for male respondents (without women’s characteristics) 8](#_Toc152683344)

[Table S5: Multivariate probit estimation of the determinants of wheat traits for women respondents (without men’s characteristics) 11](#_Toc152683345)

[Table S6: Correlation coefficient (Balanced sample) 14](#_Toc152683346)

Table S1: Trait preferences between men and women respondents (Balanced 984 men and women respondents)

| Traits | Men | Women | Difference |
| --- | --- | --- | --- |
| High yield | 81.20 | 81.10 | 0.10 |
|  | (1.25) | (1.25) | (1.76) |
| Good adaptation | 74.90 | 73.88 | 1.02 |
|  | (1.38) | (1.40) | (1.97) |
| High straw yield | 54.17 | 45.43 | 8.74*** |
|  | (1.59) | (1.59) | (2.25) |
| Disease resistance | 67.58 | 63.11 | 4.47** |
|  | (1.49) | (1.54) | (2.14) |
| Bold grain | 48.78 | 46.85 | 1.93 |
|  | (1.59) | (1.59) | (2.25) |
| Good test & cooking quality | 26.02 | 31.81 | -5.79*** |
|  | (1.40) | (1.48) | (2.04) |

NB: Observations are from 984 men and women from the same household. Standard error in parenthesis. ***, **, and * shows significance level at 1%, 5%, and 10%, respectively.

Table S2: Trait preferences difference by rainfall endowment and gender (Balanced sample)

|  | Men respondents | | | Women respondents | | |
| --- | --- | --- | --- | --- | --- | --- |
| Traits | Rainfall deficit | Rainfall surplus | Difference | Rainfall deficit | Rainfall surplus | Difference |
| High yield | 87.72 | 80.34 | 7.37* | 85.09 | 80.57 | 4.51 |
|  | (3.08) | (1.34) | (3.88) | (3.35) | (1.34) | (3.90) |
| Good adaptation | 85.09 | 73.36 | 11.52*** | 85.96 | 72.3 | 13.67*** |
|  | (3.35) | (1.49) | (4.30) | (3.27) | (1.52) | (4.36) |
| High straw yield | 34.21 | 56.78 | -22.57*** | 25.44 | 48.05 | -22.61*** |
|  | (4.46) | (1.68) | (4.91) | (4.10) | (1.69) | (4.91) |
| Disease resistance | 58.77 | 68.74 | -9.96** | 54.38 | 64.25 | -9.87** |
|  | (4.65) | (1.57) | (4.65) | (4.68) | (1.62) | (4.80) |
| Bold grain | 41.23 | 49.77 | -8.54* | 42.11 | 47.47 | -5.37 |
|  | (4.63) | (1.70) | (4.98) | (4.64) | (1.69) | (4.97) |
| Good taste & cooking quality | 23.68 | 26.32 | 2.63 | 41.23 | 30.57 | 10.65** |
|  | (3.99) | (1.49) | (4.37) | (4.63) | (1.56) | (4.63) |

NB: Observations are from 984 male and female respondents from the same household. Standard error in parenthesis. ***, **, and * shows significance level at 1%, 5%, and 10%, respectively.

Table S3: Multivariate probit estimation of the determinants of wheat traits for women respondents (Balanced sample)

|  | (1) | (2) | (3) | (4) | (5) | (6) |
| --- | --- | --- | --- | --- | --- | --- |
| Explanatory variables | High yield | Good adaptation | High straw yield | Disease resistant | Bold grain | Good taste and cooking quality |
| Age of man respondent *(years)* | -0.010 | -0.012** | -0.005 | 0.006 | -0.012** | -0.010* |
|  | (0.006) | (0.006) | (0.005) | (0.006) | (0.005) | (0.006) |
| Age of woman respondent *(years)* | 0.003 | 0.011 | 0.002 | 0.000 | 0.009 | 0.009 |
|  | (0.007) | (0.007) | (0.006) | (0.007) | (0.006) | (0.007) |
| Education of man respondent *(years of schooling)* | -0.010 | -0.014 | 0.010 | -0.012 | 0.008 | -0.002 |
|  | (0.010) | (0.009) | (0.008) | (0.009) | (0.008) | (0.009) |
| Education of woman respondent *(years of schooling)* | 0.001 | -0.021 | -0.046*** | 0.005 | -0.040** | 0.005 |
|  | (0.020) | (0.018) | (0.018) | (0.019) | (0.018) | (0.018) |
| Household size *(persons)* | -0.033 | -0.044* | -0.025 | 0.009 | -0.043** | -0.038* |
|  | (0.025) | (0.023) | (0.022) | (0.023) | (0.021) | (0.023) |
| Farm size *(ha)* | 0.028 | 0.063 | 0.052 | 0.008 | 0.072* | -0.012 |
|  | (0.047) | (0.045) | (0.039) | (0.042) | (0.038) | (0.042) |
| Livestock owned (*TLU*) | 0.007 | 0.008 | 0.006 | -0.014 | -0.011 | -0.012 |
|  | (0.012) | (0.011) | (0.010) | (0.011) | (0.010) | (0.011) |
| Assets owned *(Index)* | 1.580*** | 0.668* | -0.539 | -0.232 | 0.372 | 0.177 |
|  | (0.445) | (0.393) | (0.367) | (0.379) | (0.355) | (0.381) |
| Household owns mobile phone *(1=yes)* | -0.009 | 0.236** | -0.401*** | 0.563*** | 0.077 | 0.520*** |
|  | (0.129) | (0.119) | (0.112) | (0.119) | (0.109) | (0.117) |
| Household used improved wheat variety *(1=if man respondent said ‘yes’)* | -0.003 | -0.171 | 0.164 | -0.004 | 0.042 | -0.242* |
|  | (0.155) | (0.139) | (0.131) | (0.133) | (0.127) | (0.139) |
| Household used improved wheat variety *(1=if woman respondent said ‘yes’)* | -0.370** | -0.243* | -0.328** | 0.460*** | -0.007 | 0.159 |
|  | (0.164) | (0.146) | (0.138) | (0.144) | (0.134) | (0.146) |
| Number of social networks man respondent has in the village | 0.002 | 0.014 | -0.015 | -0.001 | 0.006 | 0.008 |
|  | (0.010) | (0.012) | (0.011) | (0.010) | (0.010) | (0.010) |
| Number of social networks women respondent has in the village | -0.006 | 0.015 | 0.054*** | 0.010 | -0.014 | -0.014 |
|  | (0.016) | (0.019) | (0.018) | (0.015) | (0.015) | (0.015) |
| Man respondent has had contact with government extension worker *(1=yes)* | -0.195 | -0.345** | -0.582*** | 0.162 | -0.196 | -0.632*** |
|  | (0.177) | (0.163) | (0.147) | (0.153) | (0.147) | (0.160) |
| Woman respondent had contact with government extension worker *(1=yes)* | -0.277* | -0.083 | 0.344*** | 0.363*** | 0.508*** | 0.842*** |
|  | (0.143) | (0.127) | (0.116) | (0.117) | (0.113) | (0.130) |
| Man respondent is member of farmers’group *(1=yes)* | 0.157 | -0.108 | -0.128 | 0.348*** | 0.016 | 0.248** |
|  | (0.132) | (0.118) | (0.109) | (0.119) | (0.107) | (0.113) |
| Woman respondent is member of farmers’ group *(1=yes)* | -0.150 | 0.270* | -0.237 | 0.026 | 0.014 | -0.050 |
|  | (0.165) | (0.161) | (0.145) | (0.158) | (0.142) | (0.147) |
| Man respondent is member of savings and credit group *(1=yes)* | 0.615*** | 0.109 | 0.230 | -0.246 | 0.192 | 0.291* |
|  | (0.235) | (0.186) | (0.172) | (0.174) | (0.172) | (0.175) |
| Woman respondent is member of savings and credit group *(1=yes)* | -0.125 | -0.063 | -0.054 | 0.315 | 0.476** | 0.071 |
|  | (0.250) | (0.218) | (0.202) | (0.203) | (0.203) | (0.212) |
| Man respondent is member of *Eddir* *(1=yes)* | 0.517*** | 0.653*** | 0.129 | -0.661*** | -0.186 | 0.047 |
|  | (0.162) | (0.151) | (0.148) | (0.159) | (0.142) | (0.152) |
| Woman respondent is member of *Eddir* *(1=yes)* | 0.025 | 0.141 | -0.121 | 0.174 | -0.296** | 0.066 |
|  | (0.143) | (0.128) | (0.121) | (0.126) | (0.117) | (0.124) |
| Man respondent is member of *Equb* *(1=yes)* | 0.640* | -0.091 | 0.171 | 0.068 | 0.305 | -0.273 |
|  | (0.384) | (0.297) | (0.273) | (0.286) | (0.267) | (0.282) |
| Woman respondent is member of *Equb* *(1=yes)* | -0.457 | -0.178 | 0.091 | 0.207 | -0.175 | -0.046 |
|  | (0.345) | (0.322) | (0.300) | (0.329) | (0.302) | (0.316) |
| Lagged rainfall amount received *(mm/year)* | 0.001** | 0.000* | -0.001*** | 0.000 | -0.000 | -0.000 |
|  | (0.000) | (0.000) | (0.000) | (0.000) | (0.000) | (0.000) |
| Rainfall shock *(Index)* | -0.043 | -0.020 | 0.438*** | 0.157 | 0.166* | 0.311*** |
|  | (0.115) | (0.109) | (0.102) | (0.107) | (0.097) | (0.103) |
| Rainfall surplus *(dummy: 1=yes)* | -0.249 | -0.499*** | 0.533*** | 0.024 | -0.107 | -0.499*** |
|  | (0.190) | (0.178) | (0.160) | (0.150) | (0.146) | (0.156) |
| Oromia region *(dummy: 1=yes)* | 0.633*** | 0.232 | -0.206 | 0.895*** | 0.813*** | 0.179 |
|  | (0.189) | (0.180) | (0.173) | (0.182) | (0.172) | (0.186) |
| Amhara region *(dummy: 1=yes)* | 0.312 | 0.034 | -0.381** | 0.081 | 0.225 | 0.912*** |
|  | (0.195) | (0.185) | (0.182) | (0.188) | (0.182) | (0.190) |
| Constant | 0.120 | 0.384 | 1.250** | -1.212** | -0.044 | -0.648 |
|  | (0.558) | (0.519) | (0.487) | (0.504) | (0.472) | (0.511) |
| Observations | 984 | 984 | 984 | 984 | 984 | 984 |

Note: Standard errors in parentheses. Significance levels correspond with ****p* < .01 for 1%, ***p* < .05 for 5% and, **p* < .1 for 10%.

Table S4: Multivariate probit estimation of the determinants of wheat traits for male respondents (without women’s characteristics)

|  | (1) | (2) | (3) | (4) | (5) | (6) |
| --- | --- | --- | --- | --- | --- | --- |
| Explanatory variables | High yield | Good adaptation | High straw yield | Disease resistant | Bold grain | Good taste and cooking quality |
| Age of men respondent *(years)* | -0.005 | -0.006 | -0.006 | -0.001 | 0.000 | -0.004 |
|  | (0.004) | (0.004) | (0.004) | (0.004) | (0.004) | (0.004) |
| Education of men respondent *(years of schooling)* | -0.023** | -0.023*** | 0.000 | -0.013 | -0.006 | 0.005 |
|  | (0.010) | (0.009) | (0.008) | (0.009) | (0.008) | (0.009) |
| Household size *(persons)* | -0.004 | -0.025 | -0.023 | -0.011 | -0.050** | 0.013 |
|  | (0.025) | (0.023) | (0.022) | (0.022) | (0.021) | (0.022) |
| Farm size *(ha)* | 0.023 | 0.069 | 0.050 | -0.045 | 0.081** | 0.008 |
|  | (0.048) | (0.044) | (0.040) | (0.041) | (0.039) | (0.040) |
| Livestock owned (*TLU*) | 0.014 | 0.014 | 0.024** | -0.018* | -0.002 | 0.002 |
|  | (0.012) | (0.012) | (0.011) | (0.011) | (0.010) | (0.011) |
| Assets owned (*Index*) | 1.459*** | 0.371 | -0.105 | -0.046 | 0.340 | 0.344 |
|  | (0.444) | (0.391) | (0.366) | (0.378) | (0.345) | (0.376) |
| Household owns mobile phone *(1=yes)* | 0.151 | 0.364*** | -0.497*** | 0.341*** | 0.049 | 0.328*** |
|  | (0.128) | (0.117) | (0.111) | (0.117) | (0.105) | (0.115) |
| Household used improved wheat variety *(1=if man respondent said ‘yes’)* | -0.326*** | -0.277*** | 0.017 | 0.324*** | 0.114 | 0.070 |
|  | (0.119) | (0.106) | (0.097) | (0.101) | (0.092) | (0.100) |
| Number of social networks man respondent has in the village | -0.015* | 0.009 | 0.017** | 0.029*** | 0.005 | 0.004 |
|  | (0.008) | (0.008) | (0.009) | (0.010) | (0.007) | (0.008) |
| Man respondent has had contact with government extension worker *(1=yes)* | -0.301** | -0.362** | -0.515*** | 0.250* | 0.308** | -0.036 |
|  | (0.151) | (0.141) | (0.133) | (0.134) | (0.124) | (0.135) |
| Man respondent is member of farmers’ group *(1=yes)* | 0.122 | 0.015 | -0.006 | 0.365*** | 0.056 | 0.285*** |
|  | (0.124) | (0.110) | (0.103) | (0.112) | (0.098) | (0.103) |
| Man respondent is member of savings and credit group *(1=yes)* | 0.954*** | 0.284 | 0.440*** | -0.135 | 0.592*** | 0.219 |
|  | (0.250) | (0.180) | (0.160) | (0.164) | (0.158) | (0.158) |
| Man respondent is member of *Eddir* *(1=yes)* | 0.647*** | 0.611*** | 0.239* | -0.465*** | -0.436*** | 0.044 |
|  | (0.139) | (0.132) | (0.139) | (0.146) | (0.128) | (0.136) |
| Man respondent is member of *Equb* *(1=yes)* | 0.533 | 0.051 | 0.228 | 0.842*** | 0.273 | -0.153 |
|  | (0.427) | (0.283) | (0.263) | (0.286) | (0.242) | (0.257) |
| Lagged rainfall amount received *(mm/year)* | 0.001** | 0.001** | -0.001*** | 0.000 | -0.000* | -0.001*** |
|  | (0.000) | (0.000) | (0.000) | (0.000) | (0.000) | (0.000) |
| Rainfall shock *(Index)* | -0.193* | -0.007 | 0.308*** | 0.199* | 0.154 | 0.338*** |
|  | (0.114) | (0.106) | (0.104) | (0.105) | (0.096) | (0.103) |
| Rainfall surplus *(dummy: 1=yes)* | -0.484** | -0.495*** | 0.538*** | 0.115 | 0.073 | -0.157 |
|  | (0.194) | (0.174) | (0.163) | (0.149) | (0.141) | (0.154) |
| Oromia region *(dummy: 1=yes)* | 0.504*** | 0.471*** | -0.969*** | 1.169*** | 0.704*** | -0.135 |
|  | (0.176) | (0.163) | (0.173) | (0.171) | (0.157) | (0.170) |
| Amhara region *(dummy: 1=yes)* | -0.068 | 0.249 | -1.227*** | 0.238 | 0.158 | 0.629*** |
|  | (0.190) | (0.177) | (0.188) | (0.182) | (0.172) | (0.181) |
| Constant | 0.061 | 0.051 | 2.706*** | -0.692 | -0.202 | -0.321 |
|  | (0.552) | (0.500) | (0.477) | (0.477) | (0.450) | (0.490) |
| Observations | 984 | 984 | 984 | 984 | 984 | 984 |

Note: Standard errors in parentheses. Significance levels correspond with ****p* < .01 for 1%, ***p* < .05 for 5% and, **p* < .1 for 10%.

Table S5: Multivariate probit estimation of the determinants of wheat traits for women respondents (without men’s characteristics)

|  | (1) | (2) | (3) | (4) | (5) | (6) |
| --- | --- | --- | --- | --- | --- | --- |
| Explanatory variables | High yield | Good adaptation | High straw yield | Disease resistant | Bold grain | Good taste and cooking quality |
| Age of woman respondent *(years)* | -0.005 | 0.002 | -0.004 | 0.006 | -0.001 | 0.001 |
|  | (0.005) | (0.005) | (0.004) | (0.005) | (0.004) | (0.004) |
| Education of woman respondent *(years of schooling)* | 0.005 | -0.021 | -0.035** | -0.004 | -0.033* | 0.007 |
|  | (0.019) | (0.018) | (0.017) | (0.018) | (0.017) | (0.017) |
| Household size *(persons)* | -0.035 | -0.051** | 0.002 | 0.014 | -0.021 | -0.022 |
|  | (0.023) | (0.022) | (0.021) | (0.022) | (0.020) | (0.022) |
| Farm size *(ha)* | 0.012 | 0.046 | 0.014 | 0.037 | 0.068* | -0.028 |
|  | (0.045) | (0.043) | (0.038) | (0.040) | (0.037) | (0.040) |
| Livestock owned (*TLU*) | 0.006 | 0.003 | 0.015 | -0.016 | -0.011 | -0.009 |
|  | (0.012) | (0.011) | (0.010) | (0.011) | (0.010) | (0.010) |
| Assets owned (*Index*) | 1.493*** | 0.373 | -0.954*** | -0.289 | 0.147 | -0.308 |
|  | (0.418) | (0.372) | (0.344) | (0.355) | (0.338) | (0.363) |
| Household owns mobile phone *(1=yes)* | 0.018 | 0.247** | -0.396*** | 0.580*** | 0.111 | 0.594*** |
|  | (0.125) | (0.117) | (0.109) | (0.116) | (0.108) | (0.113) |
| Household has used improved wheat variety *(1=if woman respondent said ‘yes’)* | -0.323*** | -0.336*** | -0.184* | 0.437*** | 0.063 | 0.021 |
|  | (0.121) | (0.110) | (0.100) | (0.107) | (0.099) | (0.103) |
| Number of social networks woman respondent has in the village | 0.009 | 0.033** | 0.033** | 0.001 | -0.015 | -0.009 |
|  | (0.013) | (0.016) | (0.014) | (0.012) | (0.012) | (0.012) |
| Woman respondent has had contact with government extension worker *(1=yes)* | -0.390*** | -0.273** | 0.103 | 0.435*** | 0.411*** | 0.464*** |
|  | (0.120) | (0.107) | (0.097) | (0.100) | (0.095) | (0.102) |
| Woman respondent is member of farmers’ group *(1=yes)* | -0.109 | 0.212 | -0.254* | 0.184 | 0.080 | 0.137 |
|  | (0.147) | (0.145) | (0.131) | (0.141) | (0.130) | (0.130) |
| Woman respondent is member of savings and credit group *(1=yes)* | 0.131 | -0.024 | 0.112 | 0.209 | 0.623*** | 0.203 |
|  | (0.226) | (0.197) | (0.187) | (0.191) | (0.189) | (0.193) |
| Woman respondent is member of *Eddir* *(1=yes)* | 0.286** | 0.408*** | -0.146 | -0.087 | -0.433*** | -0.009 |
|  | (0.118) | (0.110) | (0.105) | (0.112) | (0.105) | (0.108) |
| Woman respondent is member of *Equb* *(1=yes)* | -0.045 | -0.096 | 0.131 | 0.021 | -0.147 | -0.169 |
|  | (0.308) | (0.295) | (0.273) | (0.296) | (0.275) | (0.288) |
| Lagged rainfall amount received *(mm/year)* | 0.001*** | 0.000* | -0.001*** | 0.000 | -0.000 | -0.000 |
|  | (0.000) | (0.000) | (0.000) | (0.000) | (0.000) | (0.000) |
| Rainfall shock *(Index)* | -0.051 | -0.299*** | 0.262** | 0.031 | -0.376*** | -0.316*** |
|  | (0.120) | (0.112) | (0.103) | (0.107) | (0.103) | (0.107) |
| Rainfall surplus *(dummy: 1=yes)* | 0.723*** | 0.546*** | -0.058 | 0.584*** | 0.851*** | 0.261 |
|  | (0.174) | (0.165) | (0.157) | (0.161) | (0.160) | (0.171) |
| Oromia region *(dummy: 1=yes)* | 0.679*** | 0.378** | -0.260* | -0.369** | 0.051 | 0.808*** |
|  | (0.163) | (0.153) | (0.149) | (0.152) | (0.151) | (0.159) |
| Constant | -0.341 | -0.107 | 2.053*** | -1.043** | 0.172 | -0.769* |
|  | (0.496) | (0.458) | (0.418) | (0.439) | (0.407) | (0.439) |
| Observations | 984 | 984 | 984 | 984 | 984 | 984 |

Note: Standard errors in parentheses. Significance levels correspond with ****p* < .01 for 1%, ***p* < .05 for 5% and, **p* < .1 for 10%.

Table S6: Correlation coefficient (Balanced sample)

|  | Men | | Women | |
| --- | --- | --- | --- | --- |
| Traits | Correlation coefficients | Std error | Correlation coefficients | Std error |
| High yield and Good Adaptation | 0.457*** | (0.064) | 0.394*** | (0.061) |
| High yield and High Straw Yield | -0.032 | (0.058) | 0.008 | (0.055) |
| High yield and Disease resistance | -0.060 | (0.062) | -0.165*** | (0.059) |
| High yield and Bold grain | 0.048 | (0.058) | 0.043 | (0.056) |
| High yield and Good taste & cooking quality | 0.027 | (0.063) | 0.137** | (0.063) |
| Good adaptation and High straw yield | -0.018 | (0.054) | 0.101* | (0.053) |
| Good adaptation and Disease resistance | -0.055 | (0.057) | -0.053 | (0.056) |
| Good adaptation and Bold grain | 0.020 | (0.054) | 0.097* | (0.054) |
| Good adaptation and Good taste & cooking quality | 0.055 | (0.056) | 0.086 | (0.057) |
| High straw yield and Disease resistance | 0.060 | (0.055) | -0.033 | (0.055) |
| High straw yield and Bold grain | 0.363*** | (0.054) | 0.186*** | (0.053) |
| High straw yield and Good taste & cooking quality | 0.323*** | (0.059) | -0.068 | (0.053) |
| Disease resistance and Bold grain | 0.257*** | (0.055) | 0.192*** | (0.053) |
| Disease resistance and Good taste & cooking quality | 0.063 | (0.059) | 0.172*** | (0.056) |
| Bold grain and Good taste & cooking quality | 0.359*** | (0.058) | 0.328*** | (0.056) |

Standard errors in parentheses. Significance levels correspond with ****p* < .01 for 1%,

***p* < .05 for 5% and, **p* < .1 for 10%.
